# Practical Points

**Published:** 1894-07-14

**Authors:** 


### PRACTICAL POINTS.

[Questions are invited for this column on any point in the administration of hospitals and asylums which may prove of difficulty or interest to our readers. All communications should be marked "Practical Points," addressed to the Editor of The Hospital, 428, Strand, W.C.]

*Disinfecting Chamber.*—Referring to a query on the subject of the best disinfecting chamber for a small country hospital, which appeared in The Hospital for June 30th, page 282, our attention has been called to a description of a simple apparatus which has been found to be of much practical value by Dr. Urquhart, of James Murray's Royal Asylum, Perth, who contributed the account in question with a diagram to the *British Medical Journal* for February 7th, 1891 (page 328). Dr. Urquhart says:—"A case of scarlet fever occurred which rendered it necessary that we should possess some means of practical disinfection. Reference to the discussion on this subject at the recent annual meeting at Leeds made it clear that steam at low pressure gave unequivocal and satisfactory results. We therefore connected a large wine cask with the steam boiler in such a manner that the dry steam entered at the upper part and escaped at the bottom. A gauge registered the pressure at about three pounds, and a thermometer inserted into the outlet pipe showed a minimum temperature of 212 degrees. In order that the penetrating quality of the steam under these circumstances might be tested, a tightly rolled blanket was placed in the barrel. In twenty minutes it was removed, and the minimum thermometer which had been wrapped in the innermost folds of the blanket, was found to have stood at 212 deg. The apparatus stands in the open air at a distance from the boiler, and it was therefore necessary to dry the steam by providing a V-tube of calculated length to maintain the pressure of steam by a column of condensed water, and to act as a safety valve. The practical result of the employment of this inexpensive and easily provided apparatus was that the articles disinfected suffered very little harm. For instance, a yellow-covered novel showed only slight blisters on the boards after twenty minutes in the steam. The chemical disinfectant used was creolin."

